# Characteristics of myocardial work during exercise stress echocardiography in healthy adults

**DOI:** 10.3389/fcvm.2025.1511464

**Published:** 2025-02-28

**Authors:** Liwei Huang, Luwei Ye, Hongmei Zhang, Qingfeng Zhang, Geqi Ding, Chunmei Li, Yan Deng, Lixue Yin, Yi Wang

**Affiliations:** ^1^ Department of Cardiovascular Ultrasound and Non-invasive Cardiology, Sichuan Provincial People’s Hospital, University of Electronic Science and Technology of China, Chengdu, China; ^2^ Ultrasound in Cardiac Electrophysiology and Biomechanics Key Laboratory of Sichuan Province, Sichuan Provincial People’s Hospital, University of Electronic Science and Technology of China, Chengdu, China

**Keywords:** myocardial work, exercise echocardiography, peak positive strain, force, healthy adults

## Abstract

**Background:**

Non-invasive myocardial work (MW) is a more precise parameter for evaluating left ventricular (LV) systolic function. However, studies examining sex-based differences in MW during exercise stress echocardiography (SE) in healthy individuals are scarce. Previous research has shown that global work efficiency (GWE) decreases following exercise.

**Objectives:**

To characterize sex-based differences in MW during exercise SE in healthy adults and to explore the factors influencing the decline in GWE post-exercise.

**Methods:**

The study enrolled 200 healthy adults, all of whom underwent echocardiographic assessments both at rest and immediately after completing a symptom-limited treadmill stress test. We measured LV volume, ejection fraction (EF), force, peak positive strain (PPS), global work index (GWI), global constructive work (GCW), global wasted work (GWW), and GWE at rest and post-exercise.

**Results:**

GWI, GCW, and GWW increased, while GWE decreased after exercise. There were no significant differences in any of the global MW parameters between sexes at rest (all *p* > 0.05). The change in △GWE was greater in women (*p* < 0.05), but no significant differences were found in other MW reserve parameters between sexes. The multivariable linear regression analysis revealed that GWW was independently associated with PPS (*β* = 0.842, *p* < 0.0001) and force (*β* = 0.306, *p* = 0.023). Furthermore, the multivariable linear regression analysis showed that GWE was independently associated with PPS (*β* = −0.395, *p* = 0.018) and EF (*β* = −0.236, *p* < 0.001).

**Conclusion:**

Sex had a minimal effect on MW-based LV systolic function in healthy adults. GWE decreased post-exercise, and both PPS and force were independently associated with GWW. These findings suggest that higher contractility is achieved at the cost of increased wasted work, which subsequently leads to a decrease in mechanical efficiency.

## 1 Introduction

The assessment of myocardial function is a critical component of routine clinical practice. Myocardial strain provides additional insights into cardiac performance beyond traditional parameters of left ventricular (LV) systolic function (1). However, strain does not reflect myocardial work (MW) and oxygen consumption, as it does not adjust for loading conditions. MW, which incorporates both deformation and load into the analysis, has been proposed as an alternative tool for evaluating LV myocardial systolic function. MW assessment was initially calculated using invasive pressure measurements, which limited its widespread application in clinical practice (2, 3). Russell et al. (4) proposed a method for the non-invasive estimation of regional and global MW through the analysis of pressure-strain loops (PSLs). To date, some studies have used this technique to analyze the reference ranges of MW in normal adults (5, 6). The technique has also been employed to analyze LV systolic function in patients with coronary artery disease and cardiac amyloidosis (7–9). However, studies on MW parameters and sex differences during exercise stress echocardiography (SE) in normal adults are scarce. Borrie et al.’s study found that global work efficiency (GWE) decreased after exercise in healthy subjects, but their sample size was small and sex differences were not explored (10). The aims of this study were to characterize sex differences in MW changes during exercise SE and explore the factors influencing the decline in GWE after exercise.

## 2 Methods

### 2.1 Study population

**Figure 1:**
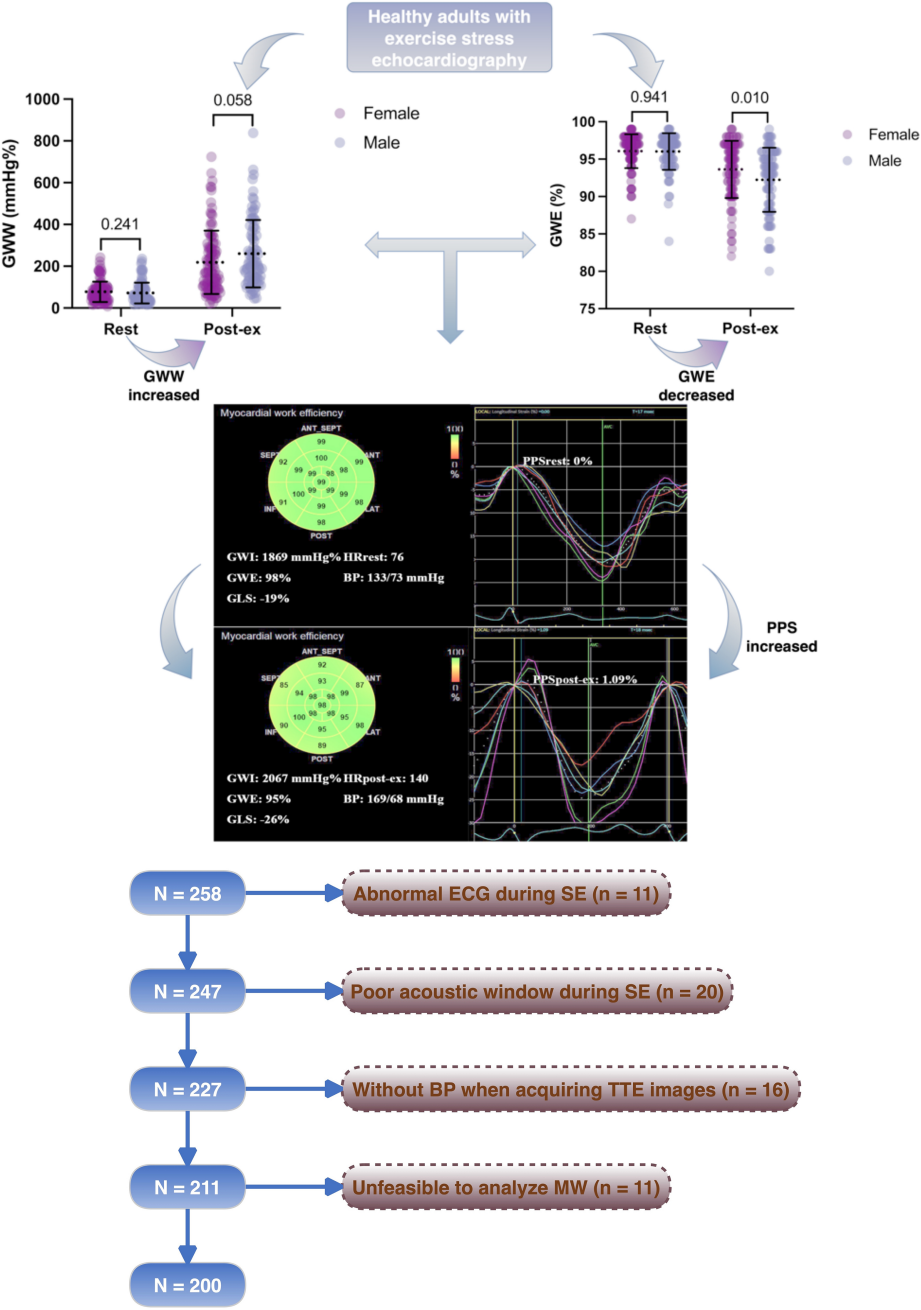
Flowchart of this study.

In this cross-sectional study, we prospectively enrolled 258 healthy volunteers between March 2018 and December 2022. The normal participants had blood pressure values within the normal reference range; normal resting and SE results; negative coronary computed tomography (CT) or angiography findings; no history of medication use; no history of hypertension, diabetes, or cardiac diseases; and were non-smokers. Based on these criteria, 58 subjects were excluded for the following reasons: electrocardiographic abnormalities during SE (11 subjects), poor acoustic window during SE (20 subjects), lack of blood pressure measurement during transthoracic echocardiography (16 subjects), and inability to analyze MW (11 subjects) (Figure 1).

All subjects underwent clinical examinations, resting 12-lead electrocardiography (ECG), and comprehensive transthoracic echocardiography both at rest and immediately following maximal treadmill stress testing. The clinical examination included measurements of height, weight, body mass index (BMI), and resting blood pressure. Body surface area (BSA) was calculated using the Du Bois formula (11). The normal adults were categorized into male and female groups for subsequent analysis. All subjects were over 18 years of age and were included after providing informed written consent in accordance with the principles of the Declaration of Helsinki. The study was approved by the local scientific ethics committee of Sichuan Provincial People's Hospital.

### 2.2 Echocardiographic data acquisition

All subjects underwent standard transthoracic echocardiography using a commercially available ultrasound system, Vivid E95 equipped with an M5S 3.5-MHz transducer (GE Vivid E95, Vingmed Ultrasound, Horten, Norway), at rest and immediately after exercise in the left lateral decubitus position. Electrocardiogram-triggered echocardiographic data were acquired and digitally stored in a cine-loop format for offline analysis using EchoPAC (EchoPAC 203, General Electric Vingmed Ultrasound). At rest, all subjects underwent a comprehensive two-dimensional echocardiographic assessment in accordance with the current guidelines (12). LV end-diastolic and end-systolic volumes (LVEDV and LVESV) were measured in the apical two- and four-chamber views. LVEF was calculated using the Simpson biplane method (13). LVEDV and LVESV were indexed into BSA, presented as LV end-diastolic volume index (LVEDVI) and LV end-systolic volume index (LVESVI), respectively. Ventricular-arterial coupling (VAC) was calculated as stroke volume (SV) divided by ESV. Force was calculated as the ratio of peak systolic blood pressure (SBP) to ESV (14). Arterial elastance (AE) was calculated by dividing LV end-systolic pressure (LVESP) by SV, with LVESP estimated as arterial systolic pressure × 0.9 (15).

LV global longitudinal strain (GLS) was obtained using automated function imaging in standard two-dimensional cine loops with a frame rate >70 frames per second (16). The regional speckle area of interest was manually adjusted for optimal tracking results. The GLS was calculated at the point of peak value during systole using a 17-myocardial segment model (17).

The principles for estimating LV pressure and work have been described previously (4). Constructive work (CW) included the sum of work performed during shortening in systole and the negative work during lengthening in the isovolumetric relaxation phase. Myocardial work index (WI) denotes the total work within the area of the LV pressure-strain loop calculated from mitral valve closure to mitral valve opening. Wasted work (WW) comprises negative systolic work due to systolic lengthening and the work of shortening in the isovolumic relaxation phase. Myocardial work efficiency (WE) was calculated as the ratio of CW to (CW + WW). LV global constructive work index (GWI), global constructive work (GCW), global wasted work (GWW), and global work efficiency (GWE) were calculated as the average of myocardial WI, CW, WW, and WE across all segmental values.

All the MW parameters were analyzed both at rest and post-exercise. Event timing (mitral valve opening and closing, and aortic valve opening and closing) was manually adjusted. Each echocardiographic parameter was averaged over three consecutive cardiac cycles. From the longitudinal speckle-tracking curve, we obtained the peak positive strain (PPS) during the early systole phase (18). If no early systolic lengthening (ESL) occurred during the early systole phase, the PPS was set to zero. If ESL was present, the maximum value of the positive strain during the ESL duration was considered the PPS (Figure 1). Therefore, ESL can also be described as the PPS. △EF, △GLS, and △force were calculated as the differences in EF, GLS, and force between rest and post-exercise values. MW reserve was calculated as the difference in GWI, GCW, GWW, and GWE between rest and post-exercise values, presented as △GWI, △GCW, △GWW, and △GWE, respectively.

### 2.3 Exercise protocol

All subjects performed a symptom-limited treadmill exercise test (TMX-425, Full Vision Inc., Kansas, USA) using standard Bruce protocols (19). Participants were encouraged to exercise until exhaustion. A baseline 12-lead ECG and blood pressure were recorded, and measurements were repeated at 2-min intervals during the examination, at peak exertion, and after exercise. The maximum exercise capacity in metabolic equivalents (METs) was recorded, where 1 MET corresponds to 3.5 ml/kg/min oxygen consumption, estimated based on the protocol, speed, and grade achieved (20). Exercise duration and the ratio of peak heart rate (HR) to target HR (calculated using the 220 − age formula) were also recorded automatically.

### 2.4 Statistical analysis

All statistical analyses were performed using SPSS version 23.0 (SPSS, Armonk, NY). Continuous variables are presented as the mean ± standard deviation (SD). Skewed data are presented as median [interquartile range (IQR)]. The normality of distribution was assessed using the Kolmogorov–Smirnov test. Comparisons of continuous variables with normal distributions between groups were performed using unpaired *t*-tests, while non-normally distributed variables were analyzed using the Mann–Whitney test. The 95% confidence interval was calculated as ±1.96 SDs from the mean. Correlations between two parameters were analyzed using Pearson's correlation test. Stepwise multivariable linear regression analyses were conducted to identify independent factors associated with GWW and GWE. A *p*-value < 0.05 was considered statistically significant.

### 2.5 Intra- and inter-observer variability

GWI analyses at baseline and immediately after exercise were repeated in 10 randomly selected patients at least 4 weeks after the initial analysis. These analyses were performed by the original investigator and a second investigator, both of whom were blinded to the original measurements, to assess intraobserver and inter-observer variability. The intraclass correlation coefficient (ICC) was calculated to evaluate variability.

## 3 Results

### 3.1 Characteristics of the population and SE parameters

Resting and treadmill exercise SE were performed in 200 healthy adults (53% women; aged 47.0 ± 12.8 years; age range, 23–69 years). The clinical characteristics of the whole population are displayed in Table 1. The men had larger BSA and higher SBP and METs than the women (all *p* < 0.05).

**Table 1:** Characteristics of the population.

Parameters	Total (200)	Men (92)	Women (108)	*p*-value
Basic				
Age (years)	47.0 ± 12.8	47.0 ± 13.3	47.3 ± 12.6	0.560
Height (cm)	163.5 ± 9.4	169.3 ± 7.8	158.8 ± 4.5	<0.001
Weight (kg)	62.4 ± 12.0	68.7 ± 12.0	55.2 ± 8.1	<0.001
Body surface area (m^2^)	1.71 ± 0.23	1.85 ± 0.21	1.62 ± 0.22	<0.001
Body mass index (kg/m^2^)	22.9 ± 3.1	23.6 ± 3.1	21.8 ± 2.4	<0.001
Rest				
HR (bpm)	91 ± 15	92 ± 15	90 ± 15	0.620
SBP (mmHg)	123 ± 18	127 ± 16	119 ± 15	<0.001
DBP (mmHg)	76 ± 11	79 ± 10	74 ± 10	0.004
Peak exercise period				
HR (bpm)	160 ± 16	166 ± 16	161 ± 16	0.077
SBP (mmHg)	168 ± 20	175 ± 23	162 ± 19	<0.001
DBP (mmHg)	78 ± 12	79 ± 12	77 ± 13	0.905
Exercise time (min)	8.9 ± 1.9	9.1 ± 1.8	8.5 ± 1.8	0.030
METs	9.8 ± 1.6	9.9 ± 1.7	9.3 ± 1.7	0.029

HR, heart rate; SBP, systolic blood pressure; DBP, diastolic blood pressure; METs, metabolic equivalents.

The SE parameters are displayed in Table 2. EF, AE, VAC, force, GLS, and PPS all increased after exercise. The women had higher EF and GLS than the men both at rest and load (*p* < 0.05). The women had higher AE at rest (*p* < 0.05), but no significant difference was observed after exercise. The men had higher force than the women at rest (*p* < 0.05), but there was no significant difference after exercise. PPS was lower in the men at rest, but higher than that in the women after exercise (*p* < 0.05). △EF and △force in the men were much higher than those in the women (*p* < 0.05). In addition to differences between sexes, a comparison of the two age groups (<48 vs. ≥48 years) was also conducted (Supplementary Table S1).

**Table 2:** Stress echocardiographic parameters of left ventricular systolic function by sex in normal adults.

Parameters	Total (200)	Men (92)	Women (108)	*p*-value
Rest				
EDVI (ml/m^2^)	40.2 ± 8.6	42.1 ± 7.9	38.6 ± 7.3	0.004
ESVI (ml/m^2^)	13.9 ± 3.3	18.0 ± 5.6	13.2 ± 3.4	<0.001
EF (%)	65.4 ± 7.2	60.7 ± 11.2	65.8 ± 5.39	<0.001
Force (mmHg/ml)	5.50 ± 1.67	6.04 ± 1.76	4.88 ± 1.29	<0.001
AE (mmHg/ml)	2.85 ± 0.69	2.64 ± 0.61	3.03 ± 0.71	<0.001
VAC	1.97 ± 0.55	1.89 ± 0.47	2.03 ± 0.61	0.069
GLS (%)	20.4 ± 2.6	19.6 ± 2.7	21.1 ± 2.4	0.0003
PPS (%)	0.6 (0.3–1.3)	0.5 (0.3–1.1)	0.9 (0.3–1.4)	0.013
Peak exercise period				
EDVI (ml/m^2^)	35.7 ± 7.8	36.8 ± 8.2	37.6 ± 8.9	0.143
ESVI (ml/m^2^)	6.9 ± 2.5	6.4 ± 2.9	7.4 ± 2.9	0.009
EF (%)	80.9 ± 4.2	75.4 ± 6.5	80.2 ± 4.9	0.004
Force (mmHg/ml)	16.39 ± 6.57	17.29 ± 6.69	15.62 ± 6.39	0.075
AE (mmHg/ml)	3.60 ± 1.05	3.58 ± 0.98	3.62 ± 1.11	0.751
VAC	4.41 ± 1.54	4.41 ± 1.54	4.41 ± 1.54	>0.999
GLS (%)	24.9 ± 3.1	24.3 ± 3.1	25.4 ± 2.9	0.011
PPS (%)	1.4 (0.9–2.1)	1.6 (0.9–2.3)	1.2 (0.8–1.9)	0.043
△EF (%)	15.68 ± 5.26	17.16 ± 5.03	14.41 ± 5.15	0.002
△Force (mmHg/ml)	10.89 ± 5.03	12.41 ± 5.11	9.59 ± 4.66	0.001
△GLS (%)	5.0 (3.0–7.0)	5.0 (3.0–7.0)	4.0 (3.0–6.0)	0.325

EDVI, end-diastolic volume index; ESVI, end-systolic volume index; EF, ejection fraction; AE, arterial elastance; VAC, ventricular-arterial coupling; GLS, global longitudinal strain; PPS, peak positive strain; △, change from rest to peak.

### 3.2 MW parameters

**Figure 2:**
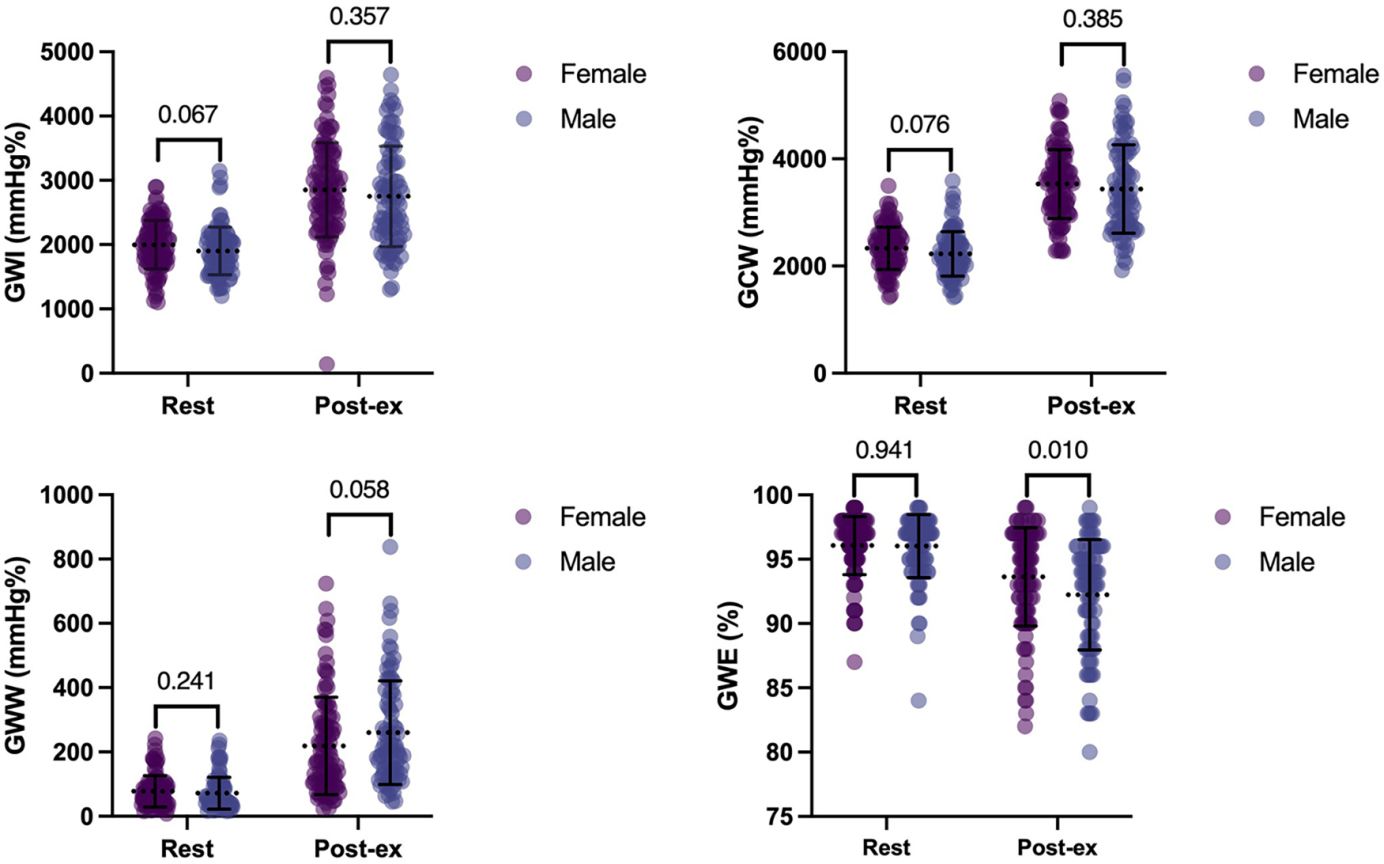
MW parameters in normal adults. There was no significant difference in all the MW parameters between sexes at rest. GWE was significantly higher in the women after exercise. GWI, global constructive work index; GCW, global constructive work; GWW, global wasted work; GWE, global work efficiency.

**Figure 3:**
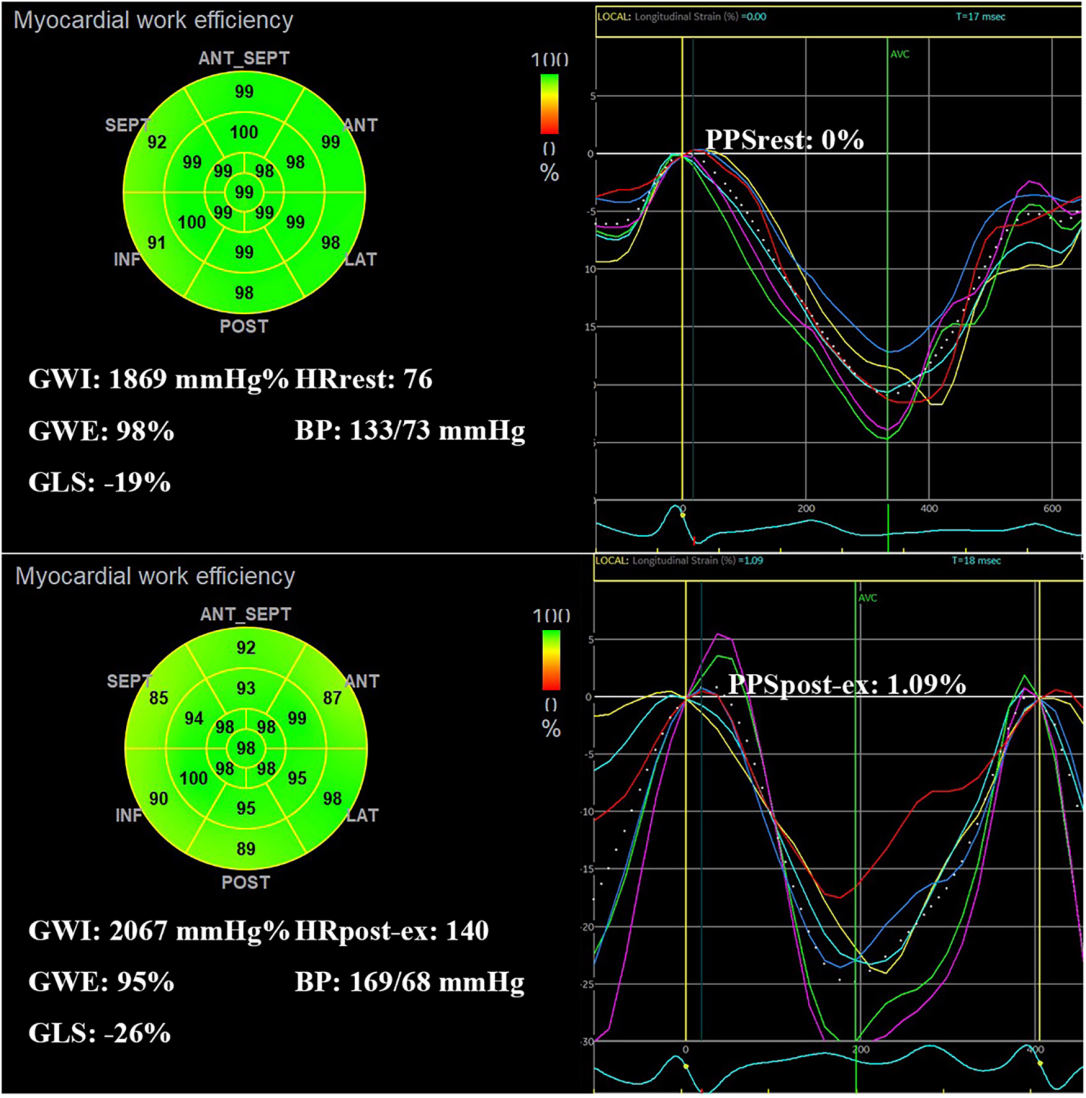
Example of a normal adult with decreased GWE and increased PPS after exercise. A bull's-eye plot of myocardial work shows GWE is 98% at rest and 95% after exercise. Time-strain curve during one cardiac cycle (white dotted line) shows PPS increased from 0% at rest to 1.09% after exercise. GWI, global constructive work index; GWE, global work efficiency; GLS, global longitudinal strain; HR, heart rate; BP, blood pressure; PPS, peak positive strain; post-ex, immediately after exercise.

MW parameters are displayed in Table 3 and Figure 2. GWI, GCW, and GWW increased, but GWE decreased after exercise (Figure 3). There was no significant difference in all global MW parameters between sexes at rest. GWE was significantly higher in the women after exercise (*p* < 0.05). △GWE was higher in the women (*p* < 0.05) but no significant differences were observed in other MW reserve parameters between sexes.

**Table 3:** Global MW parameters and MW reserve during SE.

Parameters	Total (200)	Men (92)	Women (108)	*p*-value
Rest				
GWI (mmHg%)	1,955 ± 376	1,902 ± 370	2,000 ± 376	0.067
GCW (mmHg%)	2,285 ± 406	2,229 ± 414	2,332 ± 395	0.076
GWW (mmHg%)	61 (40–97)	55 (39–94)	65 (44–99)	0.241
GWE (%)	96 ± 2	96 ± 2	96 ± 2	0.941
Peak exercise period				
GWI (mmHg%)	2,807 ± 754	2,752 ± 781	2,853 ± 732	0.357
GCW (mmHg%)	3,489 ± 730	3,438 ± 824	3,531 ± 642	0.385
GWW (mmHg%)	200 (119–326)	208 (141–368)	175 (104–291)	0.058
GWE (%)	93 ± 4	92 ± 4	94 ± 4	0.010
△GWI (mmHg%)	828 (346–1,355)	790 (341–1,442)	876 (350–1,321)	0.873
△GCW (mmHg%)	1,109 (671–1,579)	1,057 (544–1,781)	1,154 (719–1,558)	0.624
△GWW (mmHg%)	154 (76–261)	154 (81–314)	132 (67–226)	0.414
△GWE (%)	−2.0 (−5.0 to 0)	−2.0 (−6.3 to 0)	−1.0 (−4.0 to −0)	0.033

GWI, global constructive work index; GCW, global constructive work; GWW, global wasted work; GWE, global work efficiency; △, change from rest to peak.

### 3.3 The relationship between GWW, GWE, and other echocardiographic parameters

**Figure 4:**
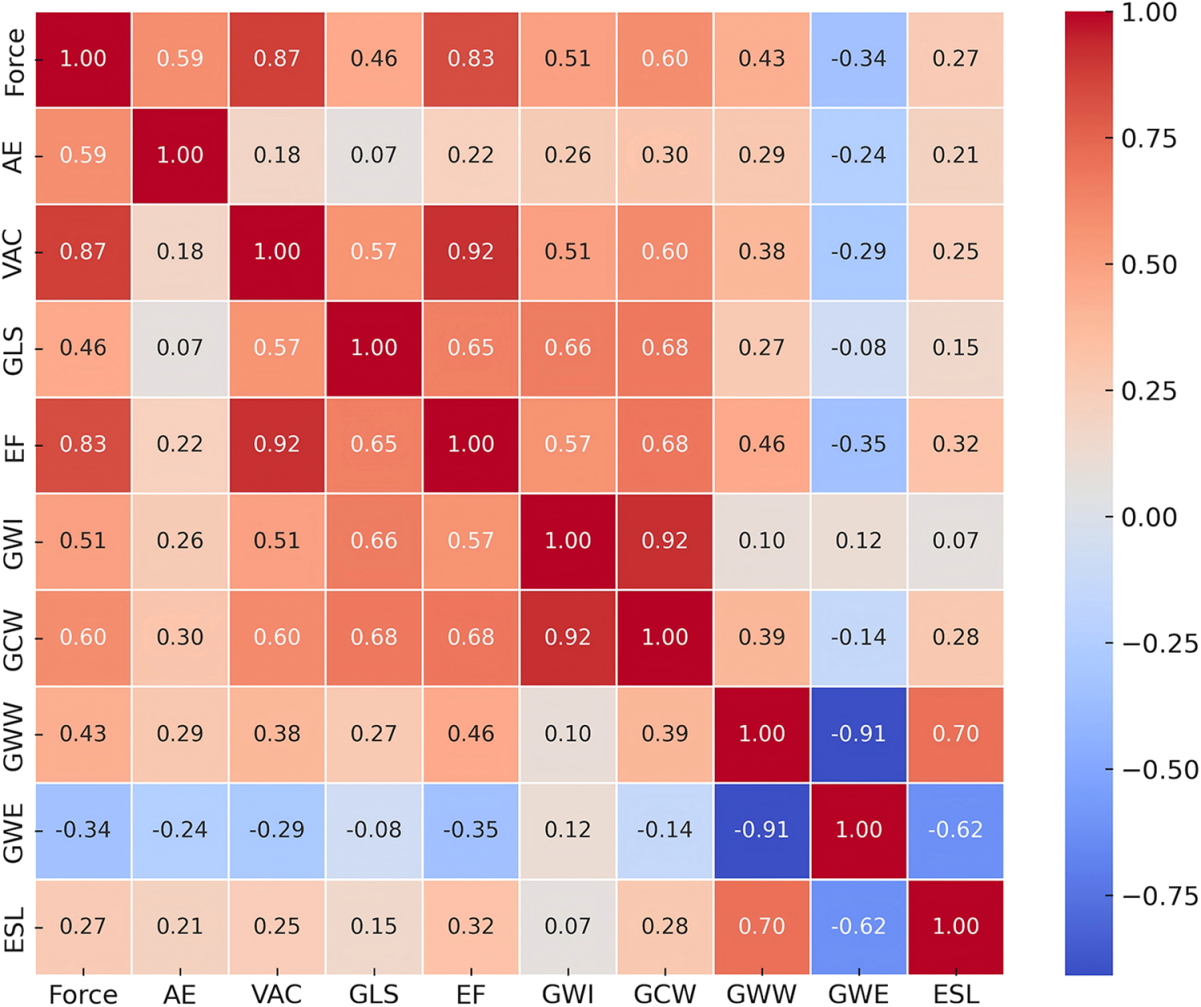
A heat map of the correlation analysis of myocardial work and other echocardiographic parameters. Red indicates a negative correlation and blue indicates a positive correlation. GLS, global longitudinal strain; EF, ejection fraction; GWI, global constructive work index; GCW, global constructive work; GWW, global wasted work; GWE, global work efficiency; ESL, early systolic lengthening; VAC, ventricular-arterial coupling; AE, arterial elastance.

Tables 4, 5 and Figure 4 show the relationship between GWW, GWE, and other echocardiographic parameters. Pearson’s correlation test showed that GWW was significantly correlated to GLS, EF, PPS, and force (all *p* < 0.0001). The multivariable linear regression analysis showed that GWW was independently associated with PPS (*β* = 0.842, *p* < 0.0001) and force (*β* = 0.306, *p* = 0.023). Pearson’s correlation test showed that GWE was significantly correlated to CI, VAC, AE, EF, PPS, and force (all *p* < 0.0001). Finally, the multivariable linear regression analysis showed that GWE was independently associated with PPS (*β* = −0.395, *p* = 0.018) and EF (*β* = −0.236, *p* < 0.001).

**Table 4:** Univariable and multivariable linear regression of global wasted work.

Parameters	*r*	*p*	*β*	*p*
GLS	0.279	<0.0001		
EF	0.431	<0.0001		
PPS	0.701	<0.0001	0.842	<0.0001
Force	0.418	<0.0001	0.306	0.023

GLS, global longitudinal strain; EF, ejection fraction; PPS, peak positive strain.

**Table 5:** Univariable and multivariable linear regression of global work efficiency.

Parameters	*r*	*p*	*β*	*p*
CI	−0.64	<0.001		
VAC	−0.623	<0.001		
AE	−0.955	<0.001		
EF	−0.139	<0.001	−0.236	<0.001
PPS	−0.843	<0.001	−0.395	0.018
Force	−0.178	<0.001		

CI, cardiac index; VAC, ventricular-arterial coupling; AE, arterial elastance; EF, ejection fraction; PPS, peak positive strain.

### 3.4 Intra- and inter-observer variability

The ICC for intraobserver variability was 0.92 for GWI at rest and 0.89 for GWI after exercise. The ICC for inter-observer variability was 0.91 for GWI at rest and 0.87 for GWI after exercise.

## 4 Discussion

The main findings of the present study can be summarized as follows: (1) there were no significant differences in most of the MW parameters between the men and women, which indicates MW is a non-sex-specific parameter to evaluate LV systolic function; (2) GWE decreased after exercise in healthy adults and PPS and force were independently associated with GWW, suggesting that higher contractility is achieved at the expense of increased wasted work, leading to decreased mechanical efficiency.

### 4.1 MW change in healthy adults during exercise

A previous study showed that SBP is an important determinant of myocardial strain (21). Increased afterload has a negative impact on strain. MW is emerging as an alternative tool for studying LV systolic function because it incorporates both deformation and load into its analysis. Russell et al. (4) demonstrated that pressure-strain loops can estimate LV performance in a non-invasive manner, deriving LV pressure curves from non-invasively acquired brachial artery cuff pressure. However, there are few reports on MW combined with treadmill exercise stress echocardiography in normal adults, and its application remains to be explored. Galli et al. (5) studied 115 normal (mean age 36.3 years, 67% men) adults and found that GWI and GCW were significantly higher in the women at rest (*p* < 0.05), but there was no significant difference in GWE between the sexes. Manganaro et al. (6) studied 226 normal adults (mean age 45 ± 13 years, 38% men). They found that only the GWE of the men at rest was significantly lower than that of the women. In our study, only the GWE of the men was significantly lower than that of the women after exercise, as other global MW parameters showed no significant difference both at rest and at load. Previous studies and this study have revealed that global MW parameters are consistent between sexes at rest, and this study further confirms that these parameters remain consistent after exercise.

It is well-known that the differences in the cardiac chamber and great arterial dimensions among the sexes are largely attributable to variations in body size (22). In this study, many of the parameters in the men were significantly higher than those in the women, which suggests that it is important to take into account the impact of sex. Previous studies have found that △GLS is significantly higher in men; however, no significant difference between the sexes was observed after adjustment for BSA (23). In this study, most of the MW reserve parameters showed no statistically significant differences. This suggests that the MW reserve is more stable than △GLS and is less influenced by sex.

### 4.2 The relationship between GWE and other echocardiographic parameters in healthy adults

When LV pressure rises during early systole, myocardial fibers with reduced contractility tend to stretch rather than shorten. This interval is referred to as the duration of ESL. It has been hypothesized that ESL occurs due to heterogeneity in the contractility profile of myocardial segments, making it a potential early marker of myocardial dysfunction (24). Previous studies have suggested that ESL may be a useful diagnostic marker for significant coronary artery disease. In addition, ESL has been shown to correlate with final infarct size following myocardial infarction (25, 26).

However, Prinzen and Lumens (27) observed that in patients with left bundle branch block (LBBB), the early-activated regions begin to shorten before aortic valve opening, thereby stretching the later-activated regions. This early systolic stretching enhances the contractile force of the late-activated regions due to the local Frank–Starling mechanism. At the same time, some studies have reported that ESL is also present in healthy adults without heart disease (24, 25). Our study also found that ESL, which can also be described as PPS, occurs in healthy adults both at rest and load. According to the definition of strain, the stretching strain during systole is represented as a positive value on the longitudinal speckle-tracking curve. The maximum value of this positive strain during the early systolic phase corresponds to PPS. Based on Russell's theory, MW is calculated from PSLs. The work performed by myocardial stretching during the systolic phase is referred to as WW. Therefore, the myocardial work during ESL duration is calculated as WW (4). Our study also found that PPS increased after exercise in healthy adults. Both previous studies and our findings indicate an increase in GWW and a decrease in GWE after loading in healthy adults. However, the underlying mechanisms and causes of these changes remain unexplained (10). Why does this phenomenon occur?

LVEF is not synonymous with LV contractility. Unlike LVEF, force is a systolic function parameter that is independent of preload and afterload (28). In both ischemic and non-ischemic hearts, preserved force is associated with a more favorable prognosis. Recently, the core protocol of SE has been enhanced by incorporating LV contractile reserve assessment based on force (14). In our study, both force and PPS increased after exercise, indicating that LV contractility improved following exercise stress. At the same time, we found that GWW was independently associated with PPS and force. This suggests that PPS may contribute to an increase in myocardial mechanical work during stress. During the early systolic phase, the myocardium initially stretches and then contracts, likely to enhance the force of myocardial contraction. As a result, LV contractility increases at the expense of elevated GWW, which subsequently leads to a reduction in myocardial mechanical efficiency.

## 5 Limitations

This is a single-center study with a moderate sample size. Further studies involving larger sample sizes are needed to explore the characteristics across different age subgroups. Second, the image quality issues inherent to speckle-tracking echocardiography were exacerbated after exercise. However, we excluded subjects without optimal image quality from the MW analysis. In addition, increased HR reduces temporal resolution and strain accuracy.

## 6 Conclusion

MW-based LV systolic function, including contractile reserve, was less influenced by sex in healthy adults. GWE decreased and GWW increased after exercise. GWW was independently associated with PPS and force, suggesting that higher contractility is achieved at the expense of increased WW, ultimately leading to reduced myocardial mechanical efficiency.

## Data Availability

The original contributions presented in the study are included in the article/Supplementary Material, further inquiries can be directed to the corresponding authors.
