# Supplementary material for: Characteristics of myocardial work during exercise stress echocardiography in healthy adults
Source: Front Cardiovasc Med. 2025 Feb 28;12:1511464. doi: 10.3389/fcvm.2025.1511464 (PMC11906847; doi:10.3389/fcvm.2025.1511464)
Supplement: Supplementary file 1 [file Table1.pdf]

SUPPLEMENTARY TABLE 1 Comparison of parameters between the two age groups.

Parameters	<48yrs (98)	≥48yrs (102)	*P*-value
**Rest**			
HR (bpm)	95±14	87±15	<0.001
SBP (mmHg)	121±16	124±15	0.139
DBP (mmHg)	76±11	77±10	0.475
EDVI (mL/m^2)	42.1±8.3	38.4±6.9	0.001
ESVI (mL/m^2)	14.8±3.8	13.2±3.2	0.002
EF (%)	64.8±5.1	65.7±5.8	0.278
Force(mmHg/mL)	5.05 ± 1.45	5.95 ± 1.75	<0.001
AE (mmHg/mL)	2.65±0.57	3.04±0.74	<0.001
VAC	1.93±0.55	2.00±0.56	0.339
GLS (%)	21.0±2.6	19.6±2.7	0.003
PPS (%)	0.9 (0.5-1.5)	0.5 (0.2-1.1)	0.004
GWI (mmHg%)	1983±380	1928±371	0.307
GCW (mmHg%)	2322±402	2248±409	0.202
GWW (mmHg%)	81±48	69±48	0.099
GWE (%)	96±2	96±2	0.292
**Peak exercise period**			
HR (bpm)	172±13	155±16	<0.001
SBP (mmHg)	166±25	169±23	0.438
DBP (mmHg)	76±13	80±13	0.023
Exercise time (min)	9.3±1.6	8.1±1.9	<0.001
METs	10.2±1.3	8.8±1.9	<0.001
EDVI (mL/m^2)	37.1±9.2	34.4±7.7	0.025
ESVI (mL/m^2)	7.4±3.3	6.5±2.5	0.032
EF (%)	80.5±4.9	81.4±5.0	0.188
Force(mmHg/mL)	15.07±5.88	17.68±6.96	0.005
AE (mmHg/mL)	3.42±1.05	3.77±1.04	0.019

VAC	4.49±1.54	4.76±1.63	0.226
GLS (%)	25.4±3.0	24.4±3.1	0.020
PPS (%)	1.2 (0.8-1.9)	1.6 (0.9-2.3)	0.184
GWI (mmHg%)	2840±747	2774±763	0.543
GCW (mmHg%)	3542±642	3436±808	0.317
GWW (mmHg%)	188 (107-308)	202 (127-347)	0.375
GWE (%)	93±4	92±4	0.110
△EF(%)	15.63±5.11	15.72±5.44	0.907
△Force (mmHg/mL)	10.02±5.49	11.73±6.43	0.045
△GLS (%)	4.0 (3.0-6.0)	5.0 (3.0-7.0)	0.493
△GWI (mmHg%)	868 (414-1307)	801 (323-1441)	0.896
△GCW (mmHg%)	1154 (719-1558)	1057 (544-1781)	0.677
△GWW (mmHg%)	121 (53-212)	148 (80-288)	0.129
△GWE (%)	-2 (-4-0)	-2 (-5-0)	0.304

HR, heart rate; SBP, systolic blood pressure; DBP, diastolic blood pressure; METs, metabolic equivalents; EDVI, end diastolic volume index; ESVI, end systolic volume index; EF, ejection fraction; VAC, ventricular-arterial coupling; AE, arterial elastance; GLS, global longitudinal strain; PPS, peak positive strain; GWI, global constructive work index; GCW, global constructive work; GWW, global wasted work; GWE, global work efficiency; △, change from rest to peak.
